# MBD2 acts as a repressor to maintain the homeostasis of the Th1 program in type 1 diabetes by regulating the STAT1-IFN-γ axis

**DOI:** 10.1038/s41418-021-00852-6

**Published:** 2021-08-21

**Authors:** Tiantian Yue, Fei Sun, Faxi Wang, Chunliang Yang, Jiahui Luo, Shanjie Rong, Haifeng Zhou, Jun Xiao, Xiaohui Wang, Qing Zhou, Ping Yang, Shu Zhang, Wen Li, Fei Xiong, Qilin Yu, Cong-Yi Wang

**Affiliations:** 1 The Center for Biomedical Research, Department of Respiratory and Critical Care Medicine, NHC Key Laboratory of Respiratory Diseases, Tongji Hospital, Tongji Medical College, Huazhong University of Sciences and Technology, Wuhan, China; 2 Department of Integrated Traditional Chinese and Western Medicine, Union Hospital, Tongji Medical College, Huazhong University of Science and Technology, Wuhan, China; 3 Department of Urology, Tongji Hospital, Tongji Medical College, Huazhong University of Science and Technology, Wuhan, China; 4 Section of Endocrinology, Department of Internal Medicine, Yale University School of Medicine, New Haven, CT USA

**Keywords:** Immunological disorders, T cells, Epigenetics, Immunological disorders

## Abstract

The methyl-CpG-binding domain 2 (MBD2) interprets DNA methylome-encoded information through binding to the methylated CpG DNA, by which it regulates target gene expression at the transcriptional level. Although derailed DNA methylation has long been recognized to trigger or promote autoimmune responses in type 1 diabetes (T1D), the exact role of MBD2 in T1D pathogenesis, however, remains poorly defined. Herein, we generated an *Mbd2* knockout model in the NOD background and found that *Mbd2* deficiency exacerbated the development of spontaneous T1D in NOD mice. Adoptive transfer of *Mbd2*^*−/*^^−^ CD4 T cells into *NOD.scid* mice further confirmed the observation. Mechanistically, Th1 stimulation rendered the *Stat1* promoter to undergo a DNA methylation turnover featured by the changes of DNA methylation levels or patterns along with the induction of MBD2 expression, which then bound to the methylated CpG DNA within the *Stat1* promoter, by which MBD2 maintains the homeostasis of Th1 program to prevent autoimmunity. As a result, ectopic MBD2 expression alleviated CD4 T cell diabetogenicity following their adoptive transfer into *NOD.scid* mice. Collectively, our data suggest that MBD2 could be a viable target to develop epigenetic-based therapeutics against T1D in clinical settings.

## Introduction

Type 1 diabetes (T1D) is a chronic autoimmune disease resulting from T cell-mediated pancreatic islet β cell destruction [1]. Therefore, strategies aimed at inhibiting autoimmune responses or inducing self-tolerance hold much better promise in facilitating β cell recovery and preventing disease progression as compared to the exogenous insulin therapy [2]. It is believed that CD4 effector T cells play a central role in immune dysregulation by producing multiple proinflammatory cytokines and orchestrating the function of cytotoxic CD8 T cells, B cells, and macrophages, which then elicit severe insulitis coupled with β cell destruction [3–5]. Among the diverse subsets of CD4 effector T cells, Th1 and Th17 are the two pivotal components contributing to T1D pathogenesis. Th1 cells are a major source of type I inflammatory cytokines, especially for IFN-γ, TNF-α, and IL-1β, which are potent to induce β cell apoptosis [6, 7]. In contrast, Th17 cells comprise a more plastic repertoire for their pathogenicity, and indeed, the diabetogenic ones are usually characterized by the conversion from non-pathogenic to pathogenic Th17 cells along with the impartment of Th1-like properties. Therefore, Th1 cells are regarded as the most critical pathogenic subset in T1D pathogenesis [3, 4].

Although genetic predisposition renders a subject with higher T1D risk, epigenetic factors linked with environmental cues also play a pivotal role to trigger autoimmune responses against pancreatic β cells [8–10]. Particularly, DNA methylation, one of the critical epigenetic mechanisms associated with the regulation of gene transcription, has been recognized to be involved in T1D pathogenesis [11, 12]. For example, studies in T1D discordant monozygotic twins identified more than 130 distinct methylated CpG sites, some of which are located in T1D susceptible regions such as HLA class II, TNF, and GAD2 [13]. The information encoded by DNA methylation is interpreted by a family of methyl-CpG-binding domain (MBD) proteins, which contains eleven known members, but only Mecp2, MBD1, MBD2, and MBD4 regulate gene transcription by directly binding to the methylated CpG DNA [14]. Upon binding to the methylated CpG DNA, they form a suppressive complex or crosstalk with nucleosome remodeling and deacetylase (NuRD) complex to achieve a coherent transcriptional program [15]. NuRD exerts versatile functions by teaming up with distinct histone deacetylases and chromatin remodeling factors, such as histone deacetylase HDAC1/2, ATP-dependent remodeling enzymes CHD3/4, histone chaperones RBBP4/7, and so on [16]. Purification and analysis of NuRD complexes revealed that MBD2 and MBD3 could form mutually exclusive NuRD complex, each recognizing methylated DNA with different affinity and selectivity. MBD2 knockout mice are viable, whereas MBD3 knockout mice are embryonically lethal [17]. Importantly, MBD2 possesses higher selectivity for methylation patterns and has been recognized in the regulation of immune response [18].

Previously, we demonstrated an indispensable role for MBD2 in Th17 differentiation and experimental autoimmune encephalomyelitis (EAE) development in C57/B6 mice [19]. In this report, we found that *Mbd2* deficiency exacerbates T1D in NOD mice. Adoptive transfer of *Mbd2*^−/−^ CD4 T cells into *NOD.scid* mice also enhanced T1D onset by promoting Th1 polarization. Mechanistically, MBD2 selectively binds to the methylated CpG DNA in the *Stat1* promoter, by which it represses STAT1-IFN-γ signaling to maintain the homeostasis of the Th1 program, and similar results were obtained in T1D patients as well. Collectively, our findings shed new light on the role of MBD2 in T1D pathogenesis, which could pave the way to develop epigenetic-based therapies against T1D in clinical settings.

## Results

### *Mbd2* deficiency exacerbates T1D development in NOD mice

**Fig. 1:**
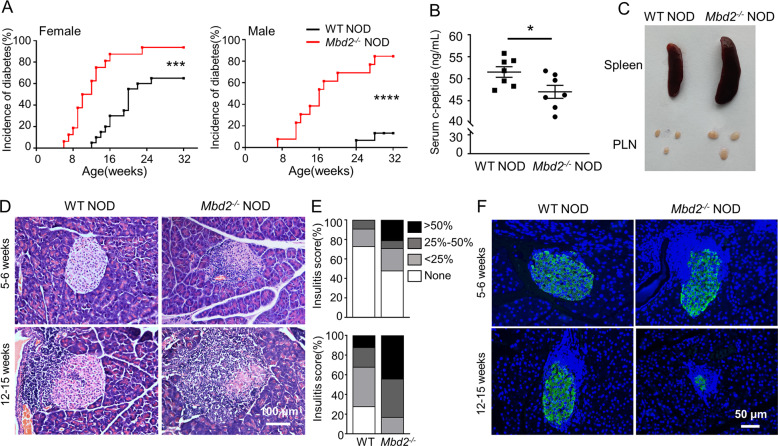
MBD2 deficiency exacerbates T1D development in NOD mice. **A** The incidence of diabetes in *Mbd2*^−/−^ NOD mice and their littermate counterparts. Left panel: female WT NOD (black line, *n* = 20) and female *Mbd2*^−/−^ NOD mice (red line, *n* = 16); right panel: male WT NOD (black line, *n* = 15) and male *Mbd2*^−/−^ NOD mice (red line, *n* = 13). **B** Plasma c-peptide levels in 8–12 weeks old pre-diabetic female WT and *Mbd2*^−/−^ NOD mice (*n* = 7 per group). **C** Representative picture of the spleen and pancreatic draining lymph nodes of 8-week-old prediabetic female WT and *Mbd2*^−/−^ NOD mice (4 mice per group). Insulitis was examined (**D**) and scored (**E**) in 5–6 and 12–15-week-old prediabetic female WT and *Mbd2*^−/−^ NOD mice respectively. Scale bar: 100 μm. The images were taken under original magnification ×200. **F** Representative results of insulin immunostaining in pancreas from prediabetic female WT and *Mbd2*^−/−^ NOD mice. Scale bar: 50 μm. The images were taken under original magnification ×200. Four mice per group were sacrificed at each time point (**D**–**F**). Diabetes incidence (**A**) was compared by log-rank test; c-peptide levels (**B**) was analyzed by the unpaired Student’s *t* test; insulitis score (**E**) was determined by *χ*^2^ test. Data are expressed as mean ± SEM. *p < 0.05, ***p < 0.001, ****p < 0.0001.

To address the role of MBD2 in T1D pathogenesis, we backcrossed *Mbd2*^−/−^ C57BL/6 mice into NOD background for more than 20 generations. The *Mbd2*^*+/−*^ NOD offspring were interbred to generate the *Mbd2*^−/−^ NOD mice. The purity of the NOD genetic background was confirmed by DNA sequencing (data not shown). We monitored the development of spontaneous diabetes in *Mbd2*^−/−^ NOD mice and littermate counterparts. The incidence of diabetes (blood glucose > 13.8 mM) was increased in both female (93.75% vs. 65%, *p* < 0.001) and male (84.62% vs. 13.33%, *p* < 0.0001) *Mbd2*^−/−^ NOD mice along with an early onset of T1D (11.47 ± 1.10 vs. 17.85 ± 1.06 weeks for females, and 14.17 ± 1.06 vs. 29.0 ± 1.0 weeks for males) (Fig. 1A). Consistently, there was a decrease of plasma c-peptide levels in the *Mbd2*^−/−^ NOD mice (Fig. 1B). The *Mbd2*^−/−^ NOD mice exhibited splenomegaly and pancreatic lymphadenopathy (Fig. 1C), suggesting a more severe autoimmune response. Next, we randomly selected pancreatic samples from nondiabetic *Mbd2*^−/−^ mice and control littermates at different disease stages to check insulitis severities. More islets with invasive insulitis (intra-islet infiltration > 50%) were observed in *Mbd2*^−/−^ NOD mice at both early prediabetic stage (5–6 weeks) and advanced diabetic stage (12–15 weeks) once compared to that of littermate controls (Fig. 1D), and the percentage of infiltrated islets was higher in *Mbd2*^−/−^ NOD group, as evaluated by insulitis scores (Fig. 1E). Moreover, the *Mbd2*^−/−^ NOD mice manifested numerous shrunk islets along with a significant reduction of insulin-positive cells, while more structured islets and insulin-secreting β cells were noted in littermate controls (Fig. 1F). We further checked the potential impact of *Mbd2* deficiency on the development of additional systemic auto-inflammatory disorders in 6–8 weeks old prediabetic NOD mice. It was interestingly noted that *Mbd2*^−/−^ NOD mice exhibited increased infiltration of lymphocytes in the salivary gland, a feature prior to the development of Sjogren’s syndrome [20], but no evident inflammatory infiltration was observed in the colon, lung, kidney, liver, or heart (Fig. S1). Together, those results indicate that loss of *Mbd2* exacerbates lymphoid infiltration and T1D onset in NOD mice.

### *Mbd2* deficiency disrupts T cell homeostasis in the PLN

**Fig. 2:**
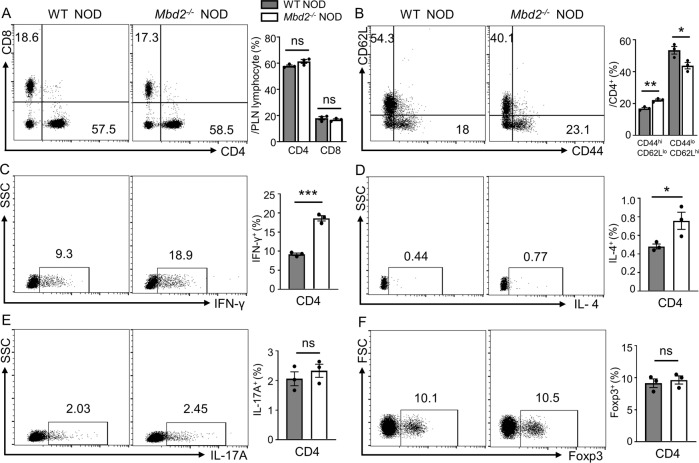
*Mbd2* deficiency enhances T cell activation and disrupts T cell homeostasis in pancreatic lymph nodes (PLNs). PLN cells from 8- to 12-week-old prediabetic WT and *Mbd2*^−/−^ NOD mice were harvested and subject to flow cytometry analysis. Frequencies of **A** CD4^+^ and CD8^+^ T cells, **B** CD4^+^ CD44^high^CD62L^lo^ and CD4^+^ CD44^lo^CD62L^high^ effector/naïve subpopulations, **C** CD4^+^ IFN-γ^+^ (Th1), **D** CD4^+^ IL-4^+^ (Th2), **E** CD4^+^ IL-17A^+^ (Th17), and **F** CD4^+^ Foxp3^+^ (Treg) subsets are shown as representative dot plot graphs. Data are expressed as mean ± SEM (three mice per group) and are representative of three independent experiments. Statistical significance was calculated by unpaired Student’s *t* test. *p < 0.05, **p < 0.01, ***p < 0.001. ns not significant.

Given the indispensable role of T cell-mediated immunity in T1D pathogenesis, we sought to examine T lymphocyte profiling in the pancreatic lymph nodes (PLN) between 8- and 12-week-old prediabetic *Mbd2*^−/−^ NOD mice and controls by flow cytometry analysis. In the PLN, although the proportions of CD4 T cells and CD8 T cells were comparable between *Mbd2*^−/−^ NOD and the control group (Fig. 2A), the *Mbd2*^−/−^ NOD mice exhibited a significantly higher proportion of CD44^hi^ CD62L^lo^ effector and lower proportion of CD44^lo^ CD62L^hi^ naïve T cells in total CD4 T cells (Fig. 2B). Notably, the frequency of CD4^+^/IFN-γ^+^ (Th1) cells was much higher in *Mbd2*^−/−^ NOD mice (Fig. 2C), and similarly, higher frequency of IFN-γ^+^ cytotoxic CD8 T cells was observed in *Mbd2*^−/−^ NOD mice (Fig. S2A). Moreover, *Mbd2*^−/−^ NOD mice displayed a modest increase of CD4^+^/IL-4^+^ (Th2) cells (Fig. 2D), but without a perceptible change in CD4^+^/IL-17A^+^(Th17) (Fig. 2E) and CD4^+^/Foxp3^+^ (Treg) subsets (Fig. 2F). To verify the systemic immune status, we checked T cell profiling in the spleen. Consistently, *Mbd2*^−/−^ NOD mice exhibited more activated CD4 T cells (Fig. S2C) and higher Th1 (CD4^+^/IFN-γ^+^) ratio than that of WT NOD mice (Fig. S2D), but no significant difference was noted in other Th subsets between the two groups (Fig. S2E–G). Collectively, those data suggest that *Mbd2* deficiency preferentially promotes the generation of IFN-γ^+^ diabetogenic T cells, Th1 cells in particular, in NOD mice, thereby breaking down the immune homeostasis to exacerbate T1D development.

### *Mbd2* deficiency enhances diabetogenicity of adoptively transferred CD4 T cells

**Fig. 3:**
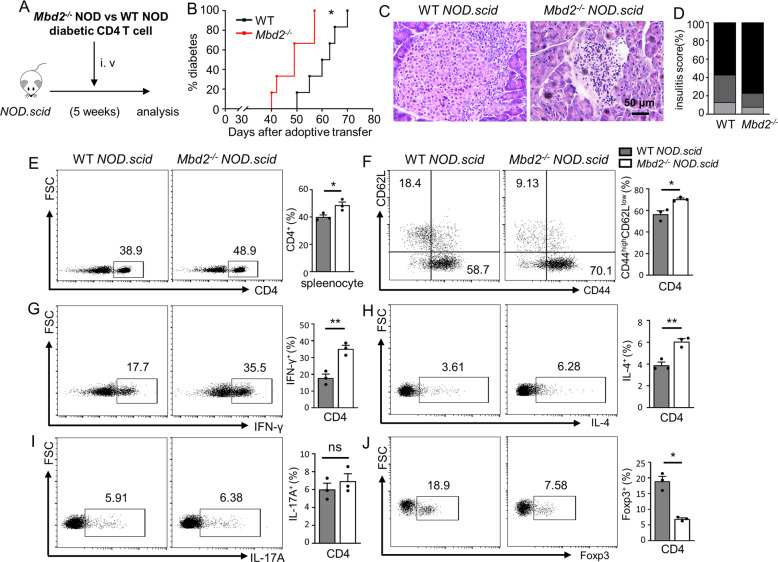
Adoptive transfer of *Mbd2* deficient diabetic CD4 T cells accelerates type 1 diabetes in *NOD.scid* mice. **A** CD4 T cells isolated from newly onset diabetic WT or *Mbd2*^−/−^ NOD mice were adoptively transferred into 5-week-old *NOD.scid* recipients (2 × 10^6^ cells/mouse) and the mice were monitored for **B** diabetes occurrence (*n* = 6 per group). Pancreases were fixed and processed for **C** H&E staining and the assessment of **D** insulitis severity (6 mice per group). Splenic cells were harvested 52 days post adoptive transfer and stained for flow cytometry. Frequencies of **E** CD4^+^, **F** CD4^+^ CD44^high^, **G** CD4^+^ IFN-γ^+^ (Th1), **H** CD4^+^ IL-4^+^ (Th2), **I** CD4^+^ IL-17A^+^ (Th17), and **J** CD4^+^ Foxp3^+^ (Treg) cell subsets are shown as representative dot plot graphs. Diabetes incidence **B** was analyzed by Log-rank test (data were pooled from two independent experiments); insulitis score **D** was determined by *χ*^2^ test, and the flow cytometry results were analyzed by the unpaired Student’s *t* test (three mice per group with two independent experiments). Data are presented as mean ± SEM. *p < 0.05, **p < 0.01. ns not significant.

To directly explore whether *Mbd2* deficiency in CD4 T cells was responsible for the observed phenotype, we adoptively transferred CD4 T cells isolated from new-onset diabetic *Mbd2*^−/−^ NOD mice and control littermates into 5-week-old *NOD.scid* (immune-deficient) recipients, followed by monitoring diabetes incidence (Fig. 3A). After the adoptive transfer, diabetes was evident by 40 days in *NOD.scid* mice receiving *Mbd2*^−/−^ CD4 T cells with all the mice developed hyperglycemia within 57 days, while a relative delay in terms of T1D development was observed in the control group (Fig. 3B). Consistently, histological analysis at day 52 following transfer revealed that the *Mbd2*^−/−^ CD4 T cell (CD4-*Mbd2*^−/−^) recipients were featured by the higher severity of islet destruction than that of control recipients (Fig. 3C, D). Indeed, the percentage of splenic CD4 T cells from CD4-*Mbd2*^−/−^ recipients was higher than the control recipients (Fig. 3E) along with an enhanced activation phenotype (Fig. 3F) and a higher Th1 ratio (Fig. 3G). The frequency of Th2 cells was also increased (Fig. 3H), while no significant change in terms of Th17 cells was noted (Fig. 3I). Moreover, the CD4-*Mbd2*^−/−^ recipients exhibited a relatively lower Treg ratio (Fig. 3J), which could be caused by the higher severity of autoimmune responses in these mice. Together, those data support the notion that MBD2 preferentially regulates Th1 cells in the T1D setting.

### MBD2 acts as an intrinsic repressor to maintain the homeostasis of Th1 program

**Fig. 4:**
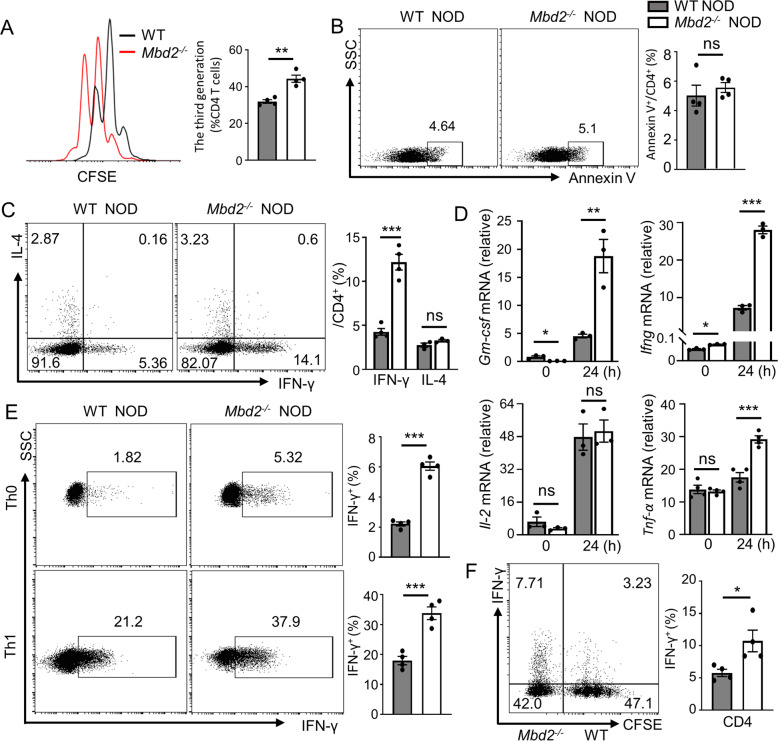
*Mbd2* deficiency leads to an intrinsically enhanced Th1 program. Splenic CD4 T cells isolated from WT and *Mbd2*^−/−^ NOD mice were activated with plate-coated anti-CD3/CD28 (10ug/ml) antibodies for 24 h. **A** The percentage of proliferated CD4 T cells was defined by CFSE assay after 3 days of culture (4 mice per group). **B** Apoptosis of T cells was determined by Annexin V staining (4 mice per group). **C** Expression of IFN-γ (4 mice per group) and IL-4 (3 mice per group) was examined in activated CD4 T cells. **D** Expression of Th1 signature genes was analyzed by real-time PCR (3–4 mice per group). **E** Naïve CD4 T cells purified from WT and *Mbd2*^−/−^ NOD splenocytes were cultured under Th0 or Th1 conditions in vitro for 3 days (4 mice per group). **F** Flow cytometry analysis of intrinsic IFN-γ expression after coculturing CFSE labeled WT naïve CD4 T cells with *Mbd2*^−/−^ naïve CD4 T cells under Th1 polarizing condition (4 mice per group). The experiments were repeated at least three times. Data are expressed as mean ± SEM. Statistical difference was analyzed by unpaired Student’s *t* test. *p < 0.05, **p < 0.01, ***p < 0.001. ns not significant.

The above results prompted us to verify the impact of MBD2 on Th1 differentiation. To this end, CD4 T cells were isolated from WT NOD and *Mbd2*^−/−^ NOD mice followed by anti-CD3/CD28 co-stimulation. Loss of *Mbd2* promoted CD4 T cell proliferation (Fig. 4A) without a perceptible impact on T cell apoptosis (Fig. 4B). Moreover, *Mbd2*^−/−^ CD4 T cells expressed greatly higher amounts of type 1 cytokines including IFN-γ (Fig. 4C), GM-CSF, and TNF-α (Fig. 4D). We then examined the impact of MBD2 on Th1 polarization. Remarkably, *Mbd2*^−/−^ CD4 T cells produced significantly higher IFN-γ even under non-polarized conditions (Th0) (Fig. 4E). The purified WT NOD naïve CD4 T cells were then labeled with CFSE and cocultured with an equal number of *Mbd2*^−/−^ NOD naïve CD4 T cells under Th1 polarizing condition. Indeed, *Mbd2*^−/−^ T cells manifested a significantly higher potency of Th1 polarization compared to the WT T cells which shared the same culture condition (Fig. 4F). Altogether, these results suggest that MBD2 serves as an intrinsic repressor to maintain the hemostasis of the Th1 program.

### *Mbd2* deficiency upregulates Th1 signature genes

**Fig. 5:**
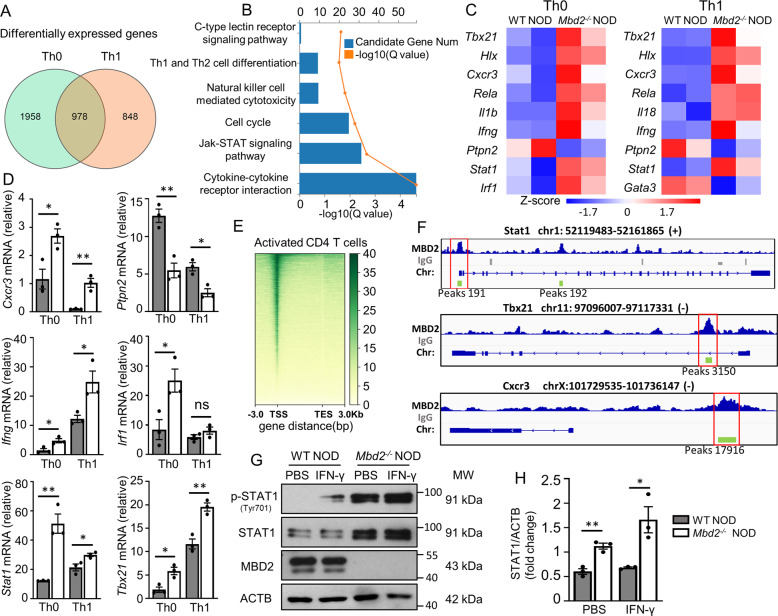
*Mbd2* deficiency upregulates Th1 signature genes. **A** RNA sequencing revealed differentially expressed genes (DEGs) of Th0 and Th1 subsets. Two biological replicates for each group, and there were two mice for each biological replicate. **B** KEGG pathway enrichment analysis on significantly altered genes between polarized WT and *Mbd2*^−/−^ Th1 cells. **C** The representative heatmap results for differentially expressed genes of WT or *Mbd2*^−/−^ Th0 and Th1 cells. **D** Real-time PCR validation for the selected Th1 signature genes in WT or *Mbd2*^−/−^ Th0 and Th1 cells (three mice per group). **E** Heatmap for the distribution of MBD2 binding sites detected by CUT&Tag assay in activated CD4 T cells. TSS transcription start site, TES transcription end site. **F** Genomic binding patterns of the *Stat1*, *Tbx21*, *Cxcr3* locus showing the MBD2-binding locations. Encircled rectangle: promoter binding sites. **G** Western blot analysis and the **H** quantitative results of STAT1 and p-STAT1 expression in isolated CD4 T cells following IFN-γ stimulation for 15 min or not (three mice per group). All in vitro studies were repeated at least three times. Data are expressed as mean ± SEM. Statistical significance was analyzed by unpaired Student’s *t* test. *p < 0.05, **p < 0.01. ns not significant.

To dissect the potential mechanisms underlying MBD2 control of the Th1 program, we firstly conducted deep RNA sequencing (RNA-seq) in Th0 and Th1 cells originated from WT NOD and *Mbd2*^−/−^ NOD mice, respectively. Ablation of *Mbd2* in Th0 and Th1 cells led to 2936 and 1826 differentially expressed genes (DEGs), and 978 of them were shared by both types of cells (Fig. 5A). KEGG analysis indicated that loss of *Mbd2* resulted in altered pathways relevant to Th1 differentiation, particularly for the Jak-STAT signaling and cytokine–cytokine receptor interaction (Fig. 5B). A set of signature genes downstream of Jak-STAT signaling such as *Ifn-γ*, *Tbx21*, *Hlx*, and *Cxcr3*, were significantly upregulated in *Mbd2*^−/−^ Th0 and Th1 cells (Fig. 5C), and quantitative polymerase chain reaction (qPCR) verified the upregulation of those Th1 signature genes in *Mbd2*^−/−^ *NOD* mice (Fig. 5D). To further define MBD2 regulated transcriptional programs, we performed a CUT&Tag sequencing assay [21] in anti-CD3/CD28 activated CD4 T cells using an MBD2 antibody. The heatmap of MBD2 CUT&Tag showed that the reads predominantly enriched in the transcription start sites (TSS) (Fig. 5E), where the CpG islands are often located. Given the observed effect of MBD2 on the Th1 program, we assessed the genes related to Th1 differentiation and found that the MBD2 binding peaks appeared in the promoter region of *Stat1*, *Tbx21*, and *Cxcr3*, as shown in the bedgraphs (Fig. 5F). In particular, *Stat1*, a master regulator for IFN-γ transcription and Th1 polarization [22], caught our attention. Exactly, the MBD2 binding peaks on *Stat1* (peaks 191: from −276 to +356 bp, the transcriptional start site as +1) covered the CpG enrichment regions (from −145 to +297 bp). As expected, the protein levels of STAT1 and p-STAT1 at baseline and after IFN-γ stimulation were significantly higher in *Mbd2*^−/−^ CD4 T cells than that of WT CD4 T cells (Fig. 5G, H). Collectively, these data pinpointed a possible role of STAT1 in mediating the upregulation of Th1 signature genes caused by *Mbd2* deficiency.

### MBD2 represses the STAT1-IFNγ axis to maintain the homeostasis of the Th1 program

**Fig. 6:**
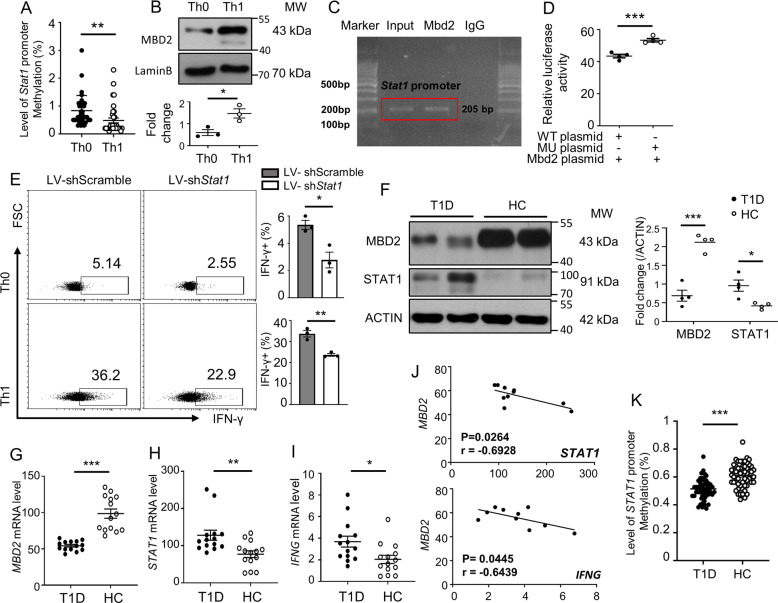
MBD2 represses the STAT1-IFNγ axis in both mouse and human CD4 T cells. **A** DNA methylation level of the predicted CpG islands of *Stat1* promoter in Th0 and Th1 cells (35 sites were included). **B** Representative western blot analysis and quantification of MBD2 expression in differentiated Th0 and Th1 cells (*n* = 3). **C** ChIP-PCR to explore the binding of MBD2 to the *Stat1* promoter region. **D** DNA methylation-dependent luciferase report assays conducted in HEK 293T cell line (*n* = 4). **E** *Mbd2*^−/−^ naïve CD4 T cells were transduced with either scramble LV-shRNA or Stat1 LV-shRNA and then cultured under Th0 and Th1 conditions to check the expression of IFN-γ (*n* = 3). **F** Representative results for Western blot analysis of MBD2 and STAT1 expression in CD4 T cells isolated from T1D patients (*n* = 4) and healthy controls (HC) (*n* = 4). Real-time PCR for analysis of **G** *MBD2*, **H** *STAT1*, and **I** *IFNG* expression in CD4 T cell isolated from T1D patients (*n* = 14) and HC (*n* = 14). **J** Correlation between *MBD2* with either *STAT1* or *IFNG* gene expression in CD4 T cells of T1D patients. **K** Result for the total methylation level of the *STAT1* promoter in CD4 T cells from T1D children (*n* = 11) and healthy controls (*n* = 8). All in vitro studies were repeated at least 3 times. Data are expressed as mean ± SEM. Statistical difference was analyzed by unpaired Student’s *t* test. *p < 0.05, **p < 0.01, ***p < 0.001. Pearson’s correlation analysis was applied for (**J**).

We next conducted studies to verify the direct regulation of MBD2 on STAT1 transcription. As MBD2 acts as a reader to interpret DNA methylome-encoded information, we check the methylation status of CpG DNA within the *Stat1* promoter. It was noted that stimulation for Th1 polarization induced the *Stat1* promoter to undergo a DNA hypomethylation in NOD-derived CD4 T cells (Fig. 6A) along with the induction of MBD2 expression (Fig. 6B). Chromatin immunoprecipitation (ChIP) was then carried out to pull down all MBD2 bound CpG DNA, and the resulting products were employed to conduct ChIP PCR using three pairs of primers flanking the CpG DNA (from −286 to +297 bp) within the *Stat1* promoter (Fig. S2A). Indeed, MBD2 is selectively bound to one of the tested regions spanning from −90 to +115 bp (Fig. 6C). DNA methylation-dependent luciferase report assays were then performed using the WT *Stat1* promoter-luciferase reporter plasmid (WT) and the CpG-mutant plasmid (MU, no CpG DNA within the binding region). As expected, co-transfection of *Mbd2* significantly suppressed the transcriptional activity of the WT *Stat1* plasmid transfected cells as compared to that of the CpG-mutant transfected counterparts (Fig. 6D). Importantly, knockdown of STAT1 by transduction of shRNA-loaded lentivirus in *Mbd2*^−/−^ naïve CD4 T cells reversed the phenotype in both Th0 and Th1 cells (Fig. 6E). Collectively, those findings suggest that MBD2 directly binds to the methylated CpG DNA within the *Stat1* promoter, by which it represses its transcription to attenuate IFN-γ signaling and Th1 program.

To further address the relationship between MBD2 induction and STAT1 transcription under a disease setting, we analyzed CD4 T cells isolated from T1D patients and healthy controls (HC). In line with our mouse data, T1D patients displayed significantly lower levels of MBD2 coupled with significantly higher levels of STAT1 (Fig. 6F). qPCR showed a consistent result at the mRNA levels (Fig. 6G, H). Particularly, a negative correlation between *MBD2* expression and *STAT1* transcription (Fig. 6J) and the expression of *IFNG*, a STAT1 downstream cytokine essential for the Th1 program (Fig. 6I, J) was observed. Since the methylated sequences were not conserved between mouse and the human within the *Stat1* promoter (Fig. S3B), we took the advantage to use the published MBD2 ChIP data (GEO: ENCSR221GAN_1 and ENCSR221GAN_2) in a human K562 cell line and found that there were MBD2 binding peaks in the promoter region of *STAT1* in K562 cells (ranging from −226 to +87 bp) (Fig. S3C). We, therefore, specifically checked the methylation status of *STAT1* promoter in CD4 T cells from T1D patients and healthy controls. We were able to detect a lower methylation level in the *STAT1* promoter region in T1D children (Fig. 6K), but were unable to obtain similar results in adult T1D patients (Fig. S3D), those patients manifested a distinctive change of methylation pattern (Fig. S3E). Altogether, our data support that Th1 polarization is featured by a DNA methylation turnover (changes in DNA methylation levels and/or patterns) in the *STAT1* promoter coupled with the induction of MBD2 expression, which then binds to the methylated CpG DNA to suppress STAT1 expression for maintaining the homeostasis of Th1 program.

### Ectopic MBD2 expression in CD4 T cells attenuates autoimmune responses in *NOD.scid* mice

**Fig. 7:**
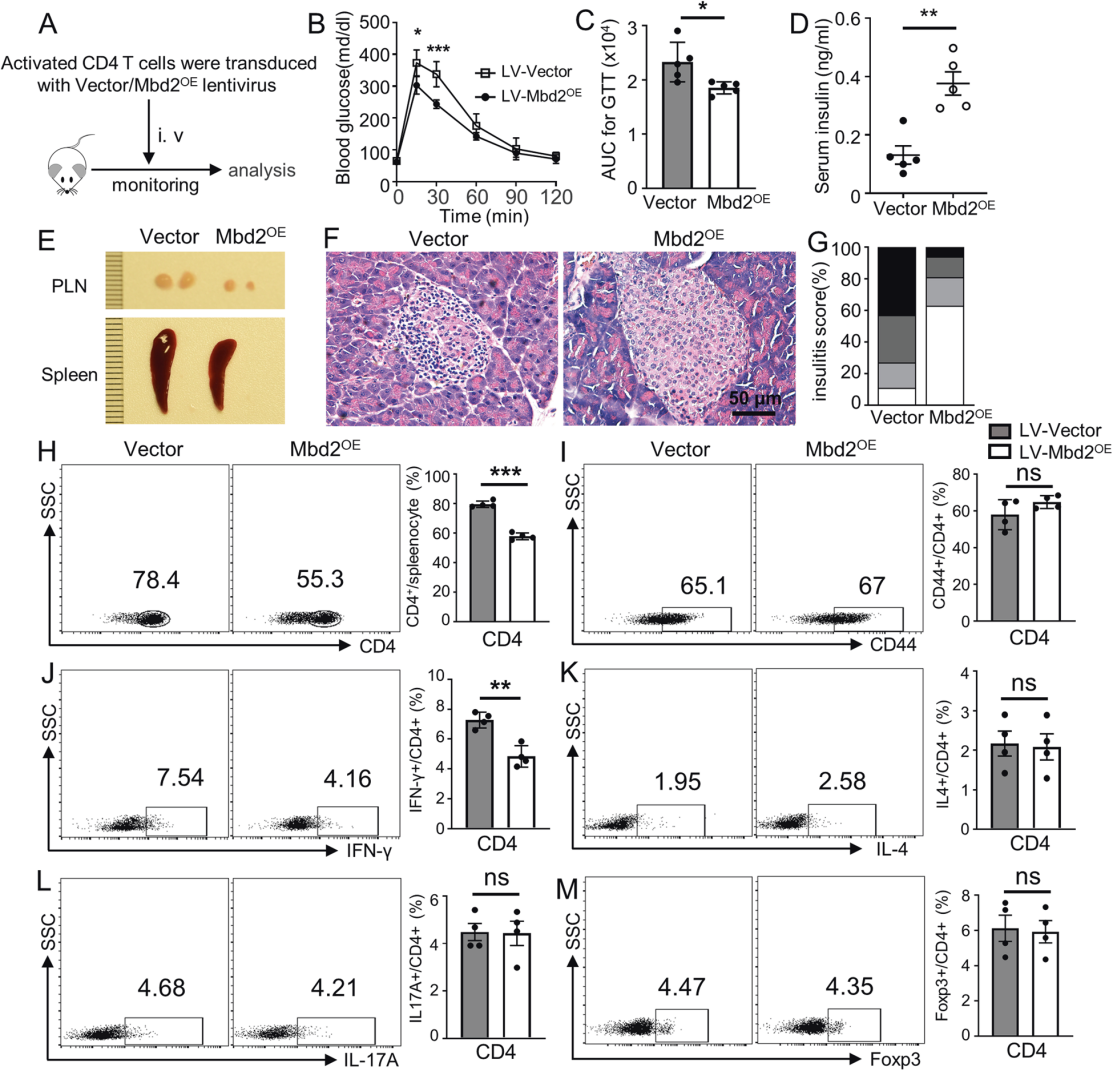
Ectopic MBD2 expression in CD4 T cells attenuates autoimmune responses in *NOD.scid* mice. **A** Purified CD4 T cells from 8-week-old nondiabetic NOD mice were transduced with either control lentivirus or lentivirus containing the MBD2 coding sequence (LV-Vector or LV-Mbd2^OE^). Cells were then adoptively transferred into 5 weeks old *NOD.scid* recipients (2 × 10^6^ cells/mouse). **B** GTT of LV-Mbd2^OE^ (*n* = 5) and LV-Vector (*n* = 5) transduced group and the respectively calculated **C** area under the curves. **D** Serum insulin levels of LV-Vector and LV-Mbd2^OE^ transduced *NOD.scid* mice (5 mice per group). **E** Images of representative PLN and spleen between LV-Vector and LV-Mbd2^OE^ recipients at 50 days post adoptive transfer (4 mice per group). Representative results for **F** H&E staining and the analysis for **G** insulitis severity between LV-Vector and LV-Mbd2^OE^ recipients at 50 days post adoptive transfer. Splenic cells were extracted at 50 days post adoptive transfer and stained for flow cytometry analysis. Frequencies of **H** CD4^+^, **I** CD4^+^ CD44^high^, **J** CD4^+^ IFN-γ^+^(Th1), **K** CD4^+^IL-4^+^(Th2), **L** CD4^+^IL-17A^+^(Th17), and **M** CD4^+^Foxp3^+^(Treg) subsets are shown as representative dot plot graphs (four mice per group). Insulitis score **G** was determined by *χ*^2^ test (5 mice per group); Statistical difference was determined by unpaired Student’s *t* test in (**B**–**D**, **H**–**M**). Data are expressed as mean ± SEM. *p < 0.05, **p < 0.01, ***p < 0.001. ns not significant.

Finally, we examined whether the adoptive transfer of *Mbd2* lentiviral transduced CD4 T cells would repress autoimmune responses in *NOD.scid* mice. CD4 T cells derived from pre-diabetic NOD mice were transduced with *Mbd2* lentivirus (LV-Mbd2^OE^) or control vector (LV-Vector) as described, and the transduction efficiency was confirmed by flow cytometry (Fig. S3A) and Western blotting (Fig. S3B). The transduced CD4 T cells (2 × 10^6^ cells per mouse) were then adoptively transferred into *NOD.scid* mice via tail vein (Fig. 7A). The mice received LV-Mbd2^OE^ transduced cells (LV-Mbd2^OE^ mice) exhibited markedly improved glucose tolerance (Fig. 7B, C) coupled with a higher level of serum insulin (Fig. 7D) as compared to that of mice received LV-Vector transduced cells (LV-Vector mice) at day 50 following the transfer, by then strong prediabetic autoimmune responses were observed. To determine the diabetogenicity of transferred CD4 T cells, the mice were sacrificed at day 50 after adoptive transfer followed by histopathological and flow cytometry analysis. Indeed, the LV-Mbd2^OE^ mice displayed smaller spleens and PLNs (Fig. 7E) along with significantly lower severity of insulitis than that of LV-Vector mice (Fig. 7F, G). Consistently, the percentage of splenic CD4 T cells from LV-Mbd2^OE^ recipients was lower (Fig. 7H) than the LV-Vector mice along with a much lower Th1 ratio (Fig. 7J), while no significant difference was noted in other Th subsets between LV-Mbd2^OE^ and LV-Vector mice (Fig. 7K–M). Collectively, those data suggest that ectopic MBD2 expression represses the Th1 program, which has the potential to repress autoimmune responses in *NOD.scid* mice, thereby attenuating the development of type 1 diabetes.

## Discussion

In the present study, we demonstrated that *Mbd2* deficiency in NOD mice exacerbates spontaneous T1D by enhancing Th1 polarization. RNA-seq verified the enhanced Th1-related gene signatures in *Mbd2*^−/−^ Th0 and Th1 cells and characterized that STAT1 may act as the most critical upstream regulator. Specifically, Th1 polarizing stimulation rendered CD4 T cells to undergo a DNA methylation turnover within the *Stat1* promoter featured by the changes of DNA methylation levels and patterns, which coupled with induction of MBD2 expression. MBD2 directly binds to the methylated CpG DNA within the *Stat1* promoter to inhibit its expression, by which it maintains the homeostasis of the Th1 program to prevent over-activation of Th1 cells. Similarly, MBD2 mRNA and protein levels were downregulated in CD4 T cells from T1D patients accompanied by the upregulation of STAT1. Adoptive transfer of *Mbd2* deficient diabetogenic CD4 T cells from NOD mice into *NOD.scid* mice exacerbated T1D development, while the transfer of MBD2 overexpressed CD4 T cells attenuated autoimmune responses. Together, our findings suggest that MBD2 directly binds to the methylated CpG DNA within the *Stat1* promoter to suppress its transcriptional activity, thereby maintaining the homeostasis of the Th1 program in CD4 T cells to prevent organ-specific autoimmunity.

T1D has been considered as a prototypical autoimmune disease where multiple immune cell types are getting involved at different disease stages. Pancreatic islet resident macrophages and CD103^+^ dendritic cells (DCs) are responsible for T1D initiation [23, 24], while CD8 T cells and B cells play a much more important role in the early pre-diabetic phase [25]. However, CD4 T cells are regarded as the major culprits considering their tremendous effect on the amplification of immune response in a clinical settings. Particularly, Th1 cells rank the most pathogenic population in mediating islet destruction. Indeed, NOD mice treated with galectin-9 (gal-9) [6] to inhibit Th1 immunity are less prone to autoimmune diabetes, and blockade of intrinsic IFN-γ signaling protects NOD mice from T1D development [7, 26, 27]. Similarly, the interferon (IFN) family contains IFN-α/β (type I) and IFN-γ (type II), both of which are strongly associated with T1D pathogenesis [28]. Type I IFN activation is induced by pattern-recognition receptors of the innate immune system [29], while IFN-γ is mainly produced by adaptive T cells or NK cells [30]. Type I IFN can activate the JAK–STAT pathway, leading to the transcription of IFN-γ and other IFN-stimulated genes (ISGs) [29], which then enhance the production of various cytokines and chemokines to orchestrate autoinflammatory response [31]. Therefore, aberrant IFN signaling is the key molecular event signifying the disruption of immune homeostasis.

Previously, we demonstrated that MBD2 implicates the pathogenesis of EAE by regulating Th17 differentiation [19]. Additional studies revealed that mice deficient in *Mbd2* manifest increased colitis severity by enhancing the activity of pro-inflammatory macrophages and DCs [32] along with overproduction of type 1 cytokines [33]. However, the exact cell types and the underlying mechanisms are yet to be fully elucidated. In this report, we employed an adoptive transfer model with *Mbd2*^−/−^ CD4 T cells to specifically highlight the suppressive role of MBD2 in Th1 differentiation and T1D pathogenesis. We sought to establish a link between pathological stimulations and epigenetic changes through MBD2 to modulate autoimmune responses in a T1D setting. Nonetheless, we noticed that there is a consistent upregulation of the Th2 cell subset in both conventional *Mbd2*^−/−^ NOD mice and those *NOD.scid* recipients transferred with *Mbd2*^−/−^ CD4 T cells. Cook et al. reported that MBD2 is required for DC activation and its capacity to elicit Th2 immunity [34], which apparently cannot explain our observed phenotype. Another issue is that MBD2 was also noted to be consistently expressed in the mouse islets, and was reduced following stressful challenges such as inflammatory cytokines (unpublished data). Therefore, the direct regulatory effect of MBD2 on the Th2 program and β cells is worthy of future investigations.

STAT1 is a key transcriptional factor involved in the regulation of Th1 lineage-specific genes [22, 35]. Indeed, gain-of-function mutation in human *STAT1* is related to the enhanced T1D susceptibility [36], but how DNA methylation affects STAT1 expression relevant to T1D pathogenesis remains less defined. Herein, we found that Th1 stimulation renders the *Stat1* promoter to undergo a DNA hypomethylation in NOD naïve CD4 T cells, and similarly, CD4 T cells from T1D patients display a change of methylation pattern in the corresponding region. ChIP-PCR confirmed that MBD2 directly binds to the methylated CpG DNA within the *Stat1* promoter, and DNA methylation-dependent promoter–reporter assays verified that MBD2 represses *Stat1* transcription following binding to the methylated DNA. Consistently, an elevated STAT1 expression along with an increased Ifn-γ production was observed in *Mbd2*^−/−^ CD4 Th0 cells, indicating a tonic suppressive effect of MBD2 on the Th1 program. It is noteworthy that a change of DNA methylation levels within the *STAT1* promoter was detected in new-onset diabetic children, but we failed to obtain a similar result in adult T1D patients, rather they manifested a change of DNA methylation pattern. In fact, T1D children were diagnosed as newly onset with a disease duration of 1.013 ± 1.011 years, while the adult patients were featured by the established T1D with an average disease duration of 4.159 ± 3.560 years (Table S1). Therefore, the discrepancy in terms of DNA methylation between two groups of patients is likely caused by the differential severity of autoimmunity. Specifically, newly onset diabetic children are generally coupled with higher severity of autoimmune immune responses, while autoimmunity would come less severe along with the progressive β cell destruction. Therefore, the initial potent autoimmune responses could induce STAT1 promoter to undergo a DNA hypomethylation, and the change of methylation levels might be gradually replaced by the change of methylation pattern with the disease progression. Since DNA methylation is a complicated process, additional possibilities such as the difference of environmental exposures cannot be completely excluded as well. During T1D progression, MBD2 recognizes and binds to the methylated CpG DNA within the *STAT1* promoter to decipher the effect of DNA methylation changes, thereby regulating the Th1 program. Given the fact that MBD2 itself does not affect DNA methylation, rather by interpreting the effect of DNA methylation changes [37], our data support that MBD2 acts as a repressor to maintain the homeostasis of the Th1 program, by which it prevents autoimmunity in the T1D setting.

Generally, T1D is diagnosed with evident symptoms in clinical settings, and by then β cell loss becomes significant to produce enough insulin to maintain glucose homeostasis. For this reason, guidelines proposed by the American Diabetes Association and Juvenile Diabetes Research Foundation were aimed to promote early diagnosis to prevent disease progression. Particularly, at stages 1 and 2 defined by the guidelines, the autoimmune attack is obvious but is yet to develop clinical symptoms, and therefore, those time windows are critical for taking interventions to preserve beta-cell function [38]. Indeed, immune therapies utilizing anti-CD3 antibody or IL-2 recombinant protein are undergoing clinical trials for T1D treatment [39, 40]. Excitingly, our studies demonstrated that ectopic MBD2 expression in CD4 T cells alleviates their diabetogenicity following adoptive transfer, as the islet-infiltrating inflammatory cells greatly reduced as compared to the controls. Nevertheless, careful verification should be tackled in future translational studies.

In summary, our findings highlighted an important role of MBD2 in T1D pathogenesis. Th1 stimulation renders the *Stat1* promoter to undergo a DNA methylation turnover featured by the changes of DNA methylation levels or patterns along with the induction of MBD2 expression, which then binds to the methylated CpG DNA within the *Stat1* promoter, by which MBD2 maintains the homeostasis of Th1 program to prevent autoimmunity. As a result, mice deficient in *Mbd2* manifest exacerbated T1D due to the loss of its repressive effect on STAT1 expression associated with the Th1 program. Since ectopic MBD2 expression alleviates CD4 T cell diabetogenicity following their adoptive transfer into *NOD.scid* mice, our data support that MBD2 related pathway could be a viable target to develop epigenetic based therapeutics against T1D in clinical settings.

## Materials and methods

### Animals

*Mbd2* knockout (*Mbd2*^−/−^) mice in the C57BL/6 background were kindly provided by Dr. Adrian Bird (Edinburgh University, UK) [41]. The *Mbd2* deficient NOD (*Mbd2*^−/−^ NOD) mice were generated by backcrossing the *Mbd2*^−/−^ C57BL/6 with NOD/ShiLtJ mice (purchased from Jackson Laboratory) for more than 20 generations, and their purity of NOD background was confirmed by DNA sequencing. *NOD.scid* mice were purchased from Beijing HFK Bioscience (Beijing, China). All mice were housed in a specific pathogen-free animal facility at the Tongji Medical College on a 12/12 h light/dark cycle. After 5 weeks of age, the NOD mice were monitored for blood glucose two times per week using an Accu-Check Advantage glucometer (Roche Diagnostics, Indianapolis, IN, USA), and classed as diabetic once two consecutive blood glucose readings were >13.8 mmol/l [42]. All protocols for animal studies were approved by the Tongji Hospital Animal Care and Use Committee (TJH-201612001) in accordance with the National Institutes of Health guidelines.

### Antibodies and reagents

Anti-mouse CD3e (#553057) and anti-mouse CD28 (#553295), recombinant mouse IL-2 (#575404) and IL-12 (#577004), and FITC-conjugated anti-mouse CD4 (#100406), PerCP-conjugated anti-mouse CD8a (#100732), PE-conjugated anti-mouse/human CD44 (#103008), APC-conjugated anti-mouse CD62L (#104412), PE/Cyanine7-conjugated anti-mouse IFN-γ (#505826), APC-conjugated anti-mouse IL-4 (#504106), APC-conjugated anti-mouse IL-17A (#506916), PE-conjugated anti-mouse IL-17A (#506904), and Alexa Fluor 647-conjugated anti-mouse Foxp3 (#126408) antibodies were purchased from the BD Biosciences and BioLegend (both San Diego, CA, USA). Anti-insulin (#4590s), anti-STAT1 (#14994S), and anti-Phospho-STAT1 (Tyr701) (#9167S) antibodies were obtained from the Cell Signaling Technology (Danvers, MA, USA). Anti-MBD2 (#ab188474) antibody was got from Abcam (Cambridge, MA, USA). The anti-Lamin B1 (#12987-1-AP) antibody was obtained from Proteintech (Wuhan, China).

### Real-time PCR and Western blot analysis

Real-time PCR and Western blot analysis were performed as previously described [43, 44]. Primers for mouse genes were: *Csf2* (forward 5′-AAC CTC CTG GAT GAC ATG CCT G-3′, and reverse 5′-AAA TTG CCC CGT AGA CCC TGC T-3′); *Ifn-γ* (forward 5′-GAT GCA TTC ATG AGT ATT GCC AAG T-3′, and reverse 5′-GTG GAC CAC TCG GAT GAG CTC-3′); *Il-2* (forward 5′-GCG GCA TGT TCT GGA TTT GAC TC-3′, and reverse 5′- CCA CCA CAG TTG CTG ACT CAT C-3′); *Tnf-α* (forward 5′-GGT GCC TAT GTC TCA GCC TCT T-3′, and reverse 5′-GCC ATA GAA CTG ATG AGA GGG AG-3′); *Cxcr3* (forward 5′-TAC GAT CAG CGC CTC AAT GCC A-3′, and reverse 5′-AGC AGG AAA CCA GCC ACT AGC T-3′); *Ptpn2* (forward 5′-AAG GTG CAG GAT ACT GTG GAG G-3′, and reverse 5′-GCC TCT GTT TCA TCT GCT GCA C-3′); *Irf1* (forward 5′-TTA GCC CGG ACA CTT TCT CTG ATG G-3′, and reverse 5′-GTC CCC TCG AGG GCT GTC AAT CTC T-3′); *Stat1* (forward 5′-TCA CAG TGG TTC GAG CTT CAG-3′, and reverse 5′-GCA AAC GAG ACA TCA TAG GCA-3′); *Tbx21* (forward 5′-CCA CCT GTT GTG GTC CAA GTT C-3′, and reverse 5′-CCA CAA ACA TCC TGT AAT GGC TTG-3′). Primers for human genes were: *MBD2* (forward 5′-AAC CCT GCT GTT TGC TTA AC-3′, and reverse 5′-CGT ACT TGC TGT ACT CGC TCT TC-3′); *STAT1* (forward 5′-CTT ACC CAG AAT GCC CTG AT-3′, and reverse 5′-CGA ACT TGC TGC AGA CTC TC-3′); and beta ACTIN (*Actb*) (forward 5′-GCA CCA CAC CTT CTA CAA TGA GC-3′, and reverse 5′-GGA TAG CAC AGC CTG GAT AGC AAC-3′). The relative expression levels for each target gene were calculated using the 2^−ΔΔCt^ method.

### Cell purification and polarization

Naïve T cells were enriched with a Naïve CD4^+^ T Cell Isolation Kit for a mouse (130-104-453; Miltenyi Biotec, Auburn, CA, USA), and CD4 T cells were enriched with either a mouse CD4 T Cell Isolation Kit (130-049-201; Miltenyi Biotec, Auburn, CA, USA) or a human CD4 T Cell Isolation Kit (130-045-101; Miltenyi Biotec, Auburn, CA, USA) according to the manufacturer’s instructions. The cells were cultured in RPMI 1640 medium (plus β-mercaptoethanol) supplemented with 10% FBS, 1% GlutaMax, 1% sodium pyruvate, and 1% Pen/Strep (all from Gibco, Shanghai, China) for further studies. For Th1 cell polarization, naïve CD4 T cells were activated with plate coated 10μg/ml anti-CD3 (145-2C11) and anti-CD28 (37.51) for 3 days in the presence of 10 ng/ml IL-2 and 10 ng/ml IL-12. For the Th0 condition, the cells were cultured with plate-coated anti-CD3 and anti-CD28 without cytokine stimulation.

### T cell proliferation assay

CD4 T cells purified from the spleens of WT and *Mbd2*^−/−^ NOD mice were stained with Cell Tracer CFSE (Life Technologies, Carlsbad, CA). The cells (1 × 10^6^/ml) were plated in the anti-CD3 and anti-CD28 coated 96-well plate in triplicates. Cell proliferation was tested after 72 h by detecting CSFE dye dilution using an mcsq10 flow cytometer.

### Adoptive transfer model

CD4 T cells were purified from splenocytes of newly onset diabetic (within one week) *Mbd2*^−/−^ and WT NOD mice using the CD4 T Cell Isolation Kit (130-049-201; Miltenyi Biotec, Auburn, CA, USA) as described earlier. CD4 T cells (2 × 10^6^/mouse) were injected through a tail vein into 5-week-old *NOD.scid* mice. All recipients were monitored for diabetes development and some of them were sacrificed at days 45 and 60 following transfer for flow cytometry analysis.

### Histological analysis

The pancreas was collected and fixed in a 10% formalin solution, followed by embedding in paraffin. Pancreatic sections were then stained with hematoxylin and eosin and were examined in a blinded fashion under a light microscope by two pathologists as reported [42, 45]. Insulitis was scored with the following grading scale: 0, no infiltration; 1, <25% infiltration of the islet; 2, 25–50% infiltration of the islet; and 3, >50% islet infiltration. More than 50 islets were scored for insulitis in each group (*n* = 4–8 mice/group).

### High throughput CUT&Tag

CUT&Tag assay was performed in Jiayin Biotechnology Ltd. (Shanghai, China) as previously described [21] with minor modifications. Briefly, nuclei were purified from anti-CD3/CD28 activated CD4 T cells as previously reported [46]. Nuclei (5 × 10^5^) were washed and incubated with Concanavalin A coated magnetic beads. The bead-bound cells were then resuspended with dig-wash buffer and a 1:50 dilution of primary MBD2 antibody (KA0694, Abnova, Taipei, Taiwan) or control IgG on a rotating platform overnight at 4 °C. Beads were washed in dig-wash buffer and incubated with a secondary antibody. After incubation, the beads were washed in dig-Hisalt buffer and incubated with proteinA-Tn5 transposome. Next, the cells were resuspended in a Tagmentation buffer. The DNA fragments were purified by phenol-chloroform and used for sequencing. Sequencing was performed in the Illumina Novaseq 6000 (Vendor’s information) using 150 bp paired-end following the manufacturer’s instructions.

### Chromatin immunoprecipitation (ChIP) assay

ChIP assays were performed as previously reported [47]. Briefly, 1 × 10^6^ WT CD4 T cells were cross-linked with 1% formaldehyde followed by sonication on ice. The MBD2-DNA complexes were next immunoprecipitated using an anti-MBD2 antibody. Normal rabbit IgG was used to determine nonspecific bindings. The eluted DNA was subjected to ChIP-PCR with indicated primers. The primers used for *Stat1* in the ChIP assay were: F1 5′-CCC TGG CTT TAG TAG CGT GAA GG-3′ and R1 5′-CTC GGC TGC TTG GCC TTC CTG GT-3′; F2 5′-CGG ACC AGG AAG GCC AAG CA-3′ and R2 5′-TCC CAA GTG GGT CTG AGG G-3′; and F3 5′-GCC GAG TCT GTC AAA GCT CCC TG-3′ and R3 5′-ATC CGG CTC CTG GCG TTC T-3′.

### Plasmid constructs and luciferase reporter assay

The peak regions for *Stat1* (from −1342 to +639 bp, the transcriptional start site as +1) were PCR amplified from mouse genomic DNA. The primers used for PCR were: F 5′-GAA CAC TGC TGG TGA GAA GAT TGA G-3′ and R 5′-CAT ACT GAG ATT CCA ACT TCC G-3′. The mutated peak region for *Stat1* was directly synthesized by the Tsingke Biological Technology (Wuhan, China), in which cytosines located in MBD2 binding CpG sites were mutated into adenosine. Both regions were cloned into the pGL3 vector as reported [14]. HEK 293T cells were transfected with either WT or MU *Stat1* plasmids along with the *Mbd2* adenovirus (043798A, Abcam Inc.) using a Lipofectamine 3000 kit (Invitrogen, USA). After 48 h of transfection, cell lysates were prepared and subjected to analysis of reporter activity using a Dual-Luciferase Reporter Assay System Kit (Promega, Madison, WI, USA). We obtained HEK 293T cells from ATCC. The cell line was routinely tested and authenticated negative for mycoplasma contamination.

### Lentiviral transduction

For *Stat1*–shRNA transduction, naïve CD4 T cells from *Mbd2*^−/−^ NOD mice were transduced with lentivirus containing the *Stat1* shRNA hairpin sequences (5′-GCT CAC TCA GAA CAC TCT GAT-3′). Briefly, naïve CD4 T cells were first cultured under Th0 or Th1 conditions as described for 24 h. The concentrated lentivirus (5 × 10^8^ Tu/ml) containing either *Stat1* shRNA or scramble shRNA (DesignGene Biotechnology, Shanghai, China) was added at MOI = 40 in the presence of 5 µg/mL Polybrene. The cells were immediately centrifuged with the virus in the plate at 1800 rpm for 2 h and then cultured at 37 °C and 5% CO_2_ for 6 h. After washing with culture medium, the cells were continually cultured under Th0 or Th1 condition as described earlier for 2 days, followed by intracellular staining for flow cytometry analysis. For ectopic MBD2 overexpression, CD4 T cells from pre-diabetic NOD mice were first activated with plate coated 10 µg/ml anti-CD3 (145-2C11) and anti-CD28 (37.51) for 24 h, and were then transduced with either control lentivirus or lentivirus containing the MBD2 coding sequence (MOI = 20) (DesignGene Biotechnology, shanghai, China) as described earlier.

### Human samples

Blood samples were obtained from T1D patients (T1D, *n* = 39) and HC (*n* = 41) with informed consent. Among the participants, there were 17 T1D children and 11 healthy children (age < 15 years). All patients with T1D had been treated with insulin since disease onset and were metabolically stable at the time of blood sampling. The patients were tested positive for T1D-specific autoantibody and 71% of patients carried the high-risk HLA diplotype alleles (DR3/DR4) [48, 49] at the time of diagnosis (Table S1). The primers used for HLA typing were listed in Table S2. None of the patients had signs of active infection, neoplasia, or other comorbidities. The healthy donors had a negative personal history of autoimmune disease. All studies in humans were conducted in accordance with the NIH guidelines and were approved by the Institutional Review Board (IRB) of Tongji Hospital.

### Statistical analysis

All in vitro studies were conducted at least three times. The Kaplan-Meier method was used for survival analysis. The Log-rank (Mantel–Cox) test was used to determine the differences in diabetes incidence between the groups. The difference in insulitis severity was determined using the *χ*^2^ test. The correlation was determined by Pearson’s correlation analysis. All other results were expressed as mean ± SEM, and their comparisons were accomplished by the Student’s *t* test with 95% CI. In all cases, *p* < 0.05 was considered statistically significant. Statistical analyses of the data were conducted using GraphPad Prism 5 software (GraphPad Software Inc., San Diego, CA, USA).

## Supplementary information

Supplementary Figure Legends

Supplementary Table 1

Supplementary Table 2

Supplementary Figure 1

Supplementary Figure 2

Supplementary Figure 3

Supplementary Figure 4.

## Data Availability

All data needed to evaluate the conclusions in this article are included in the paper and/or its supplementary information. The raw data for sequencing have been deposited in the NCBI public repository Sequence Read Archive, with an accession code PRJNA741608 (RNA-seq) and PRJNA741691(CUT&Tag). Additional data related to this paper may be requested from the authors.
