# Supplementary material for: MBD2 acts as a repressor to maintain the homeostasis of the Th1 program in type 1 diabetes by regulating the STAT1-IFN-γ axis
Source: Cell Death Differ. 2021 Aug 21;29(1):218–29. doi: 10.1038/s41418-021-00852-6 (PMC8738722; doi:10.1038/s41418-021-00852-6)
Supplement: Supplementary file 1 — Supplementary Figure Legends [file 41418_2021_852_MOESM1_ESM.docx]

**Supplementary Materials**

**Supplementary Figure 1. Histological assessment of different tissues.** Representative picture of the salivary gland, colon, lung, kidney, liver, and heart from 6-8 weeks old pre-diabetic WT and *Mbd2^-/-^* NOD mice (4 mice per group). The images were taken under original magnification x40 for salivary gland, and x100 for colon, lung, kidney, liver, heart.

**Supplementary Figure 2. *Mbd2* deficiency enhances IFN-γ^+^ cells in PLNs and spleen. (A)** Flow cytometry analysis of the IFN-γ^+^CD8^+^ T cell percentage in PLNs of WT NOD and *Mbd2^-/-^* NOD mice (3 mice per group). Splenocytes from 8-12 weeks old pre-diabetic WT and *Mbd2^-/-^* NOD mice were harvested and subject to flow cytometry analysis. Frequencies of **(B)** CD4^+^ and CD8^+^ T cells, **(C)** CD4^+^CD44^high^CD62L^lo^ and CD4^+^CD44^lo^CD62L^high^ effector/naïve subpopulations, **(D)** CD4^+^IFN-γ^+^ (Th1), **(E)** CD4^+^IL-4^+^ (Th2), **(F)** CD4^+^IL-17A^+^ (Th17), and **(G)** CD4^+^Foxp3^+^ (Treg) subsets are shown as representative dot plot graphs. Data are expressed as mean ± SEM (3 mice per group) and are representative of three independent experiments. Statistical significance was calculated by unpaired Student’s *t* test. *p<0.05, **p<0.01. ns not significant.

**Supplementary Figure 3.** The diagram for predicted CpG islands on *STAT1* promoter of **(A)** mouse and **(B)** human. **(C)** ChIP-seq analysis of MBD2 binding peaks on the *STAT1* promoter region in K562 human cell line (from published database GEO: ENCSR221GAN_1 and ENCSR221GAN_2). **(D)** Result for the total methylation level of the *STAT1* promoter in CD4 T cells from T1D adult patients (n=8) and healthy controls (n=9). **(E)** Analysis of the methylation status of *STAT1* promotor in CD4 T cells from T1D adult patients (n=8) and healthy controls (n=9).

**Supplementary Figure 4. (A)** The flow cytometry and **(B)** western blotting analysis of the transduction efficiency of CD4 T cells transduced with either vector or MBD2 overexpressed lentivirus (LV-Vector or LV-Mbd2^OE^).
