# Supplementary figures and images for: MBD2 acts as a repressor to maintain the homeostasis of the Th1 program in type 1 diabetes by regulating the STAT1-IFN-γ axis
Source: Cell Death Differ. 2021 Aug 21;29(1):218–29. doi: 10.1038/s41418-021-00852-6 (PMC8738722; doi:10.1038/s41418-021-00852-6)

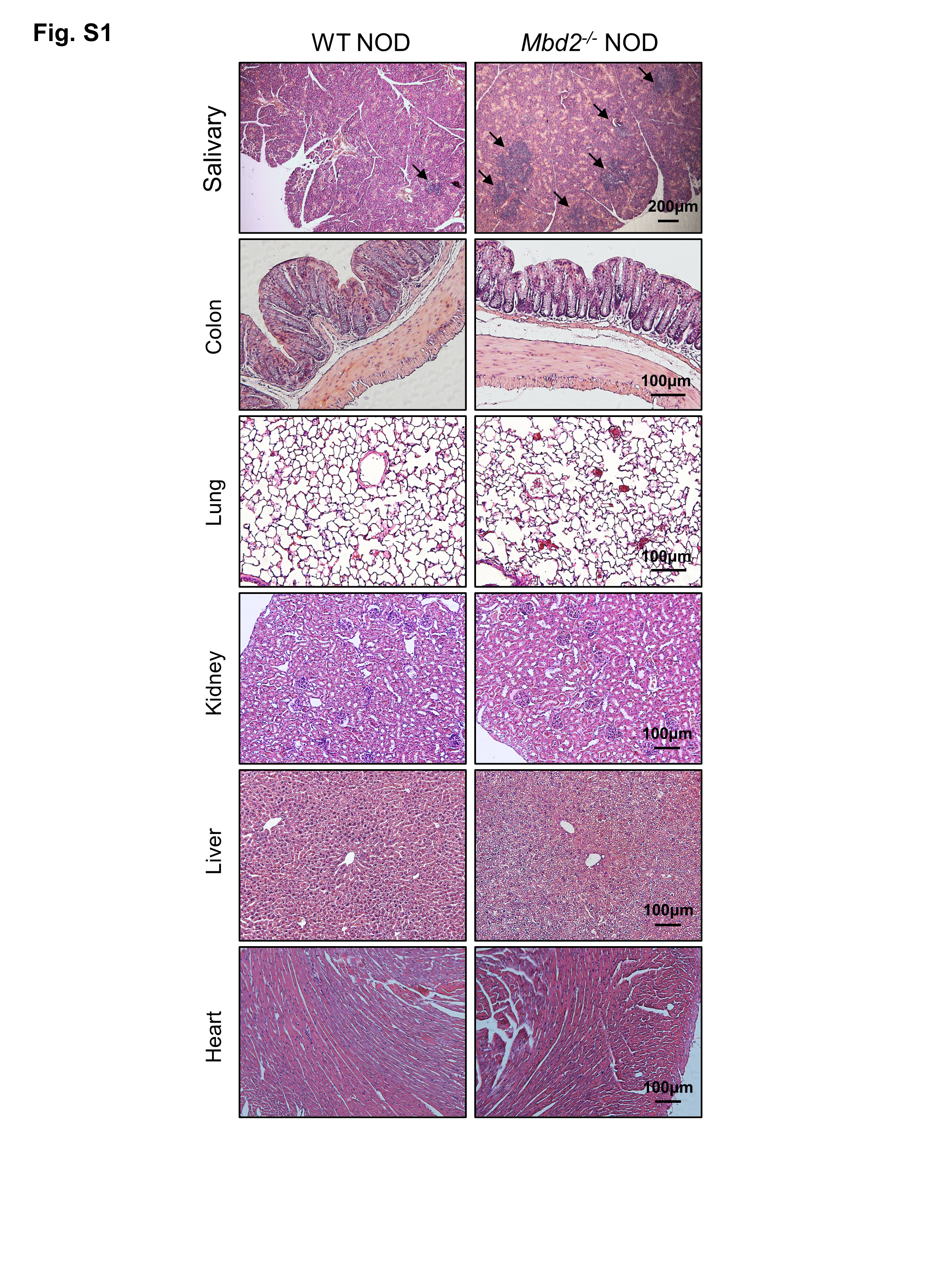

Supplement: Supplementary file 4 — Supplementary Figure 1 [file 41418_2021_852_MOESM4_ESM.tif]

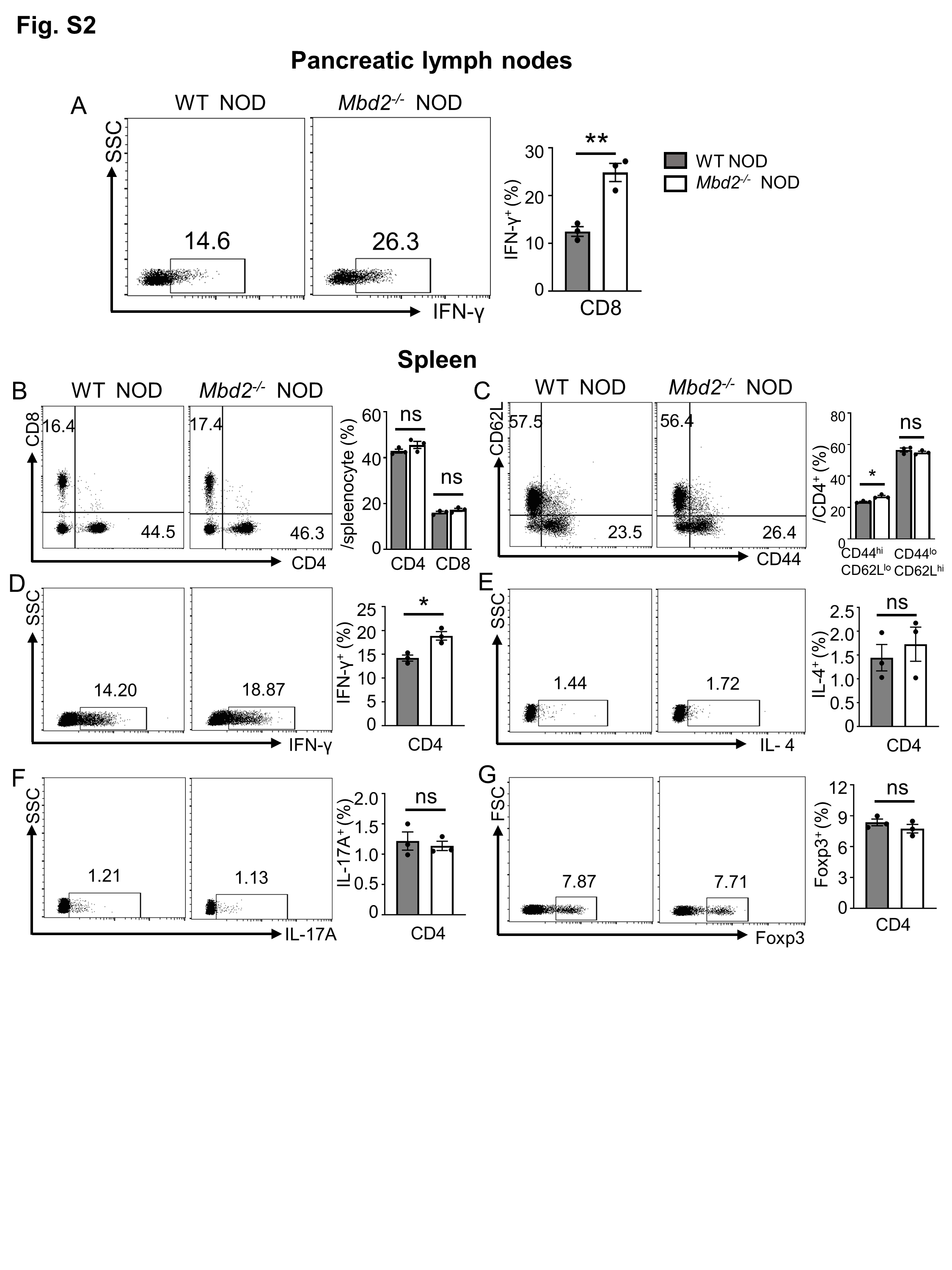

Supplement: Supplementary file 5 — Supplementary Figure 2 [file 41418_2021_852_MOESM5_ESM.tif]

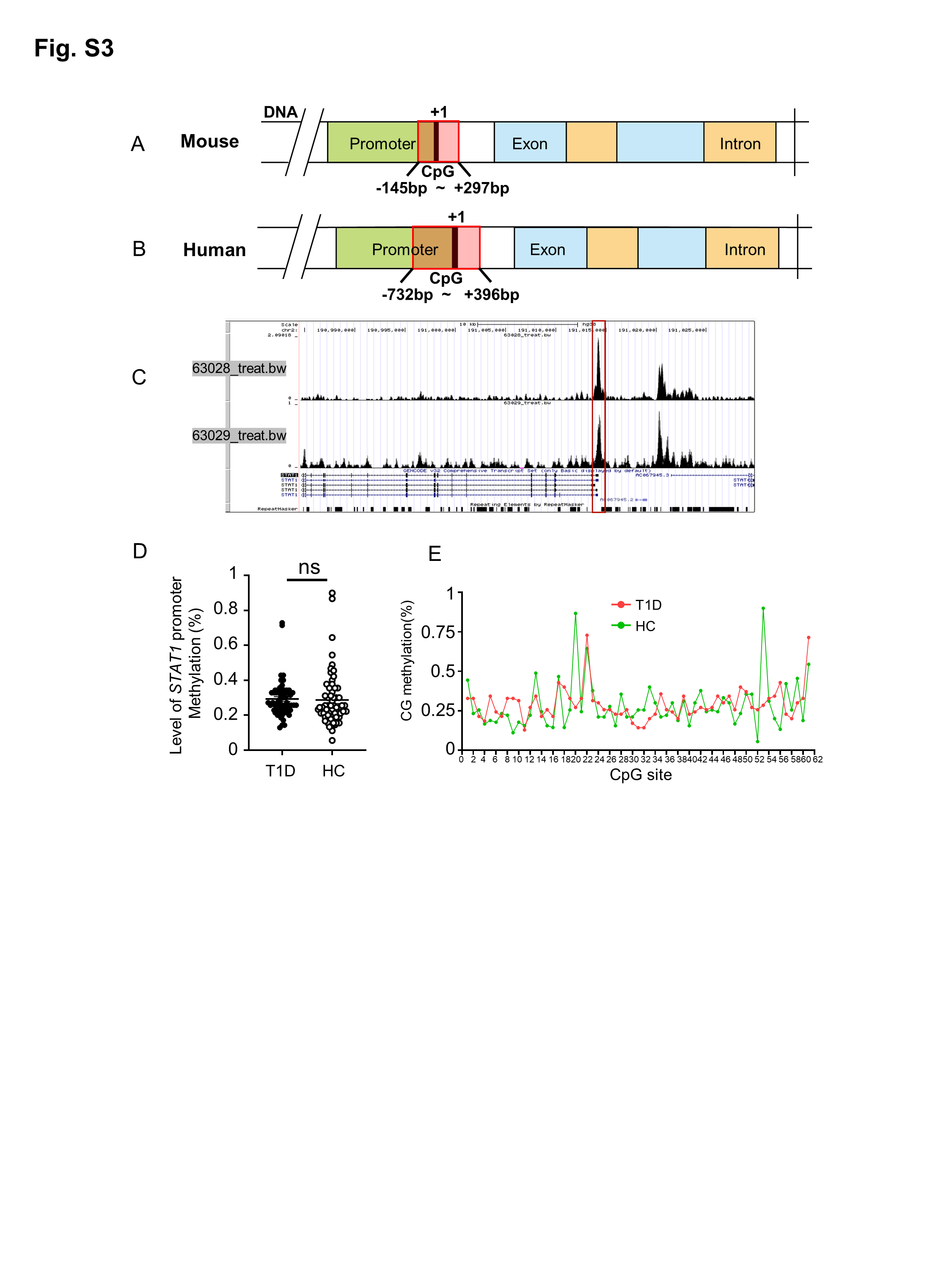

Supplement: Supplementary file 6 — Supplementary Figure 3 [file 41418_2021_852_MOESM6_ESM.tif]

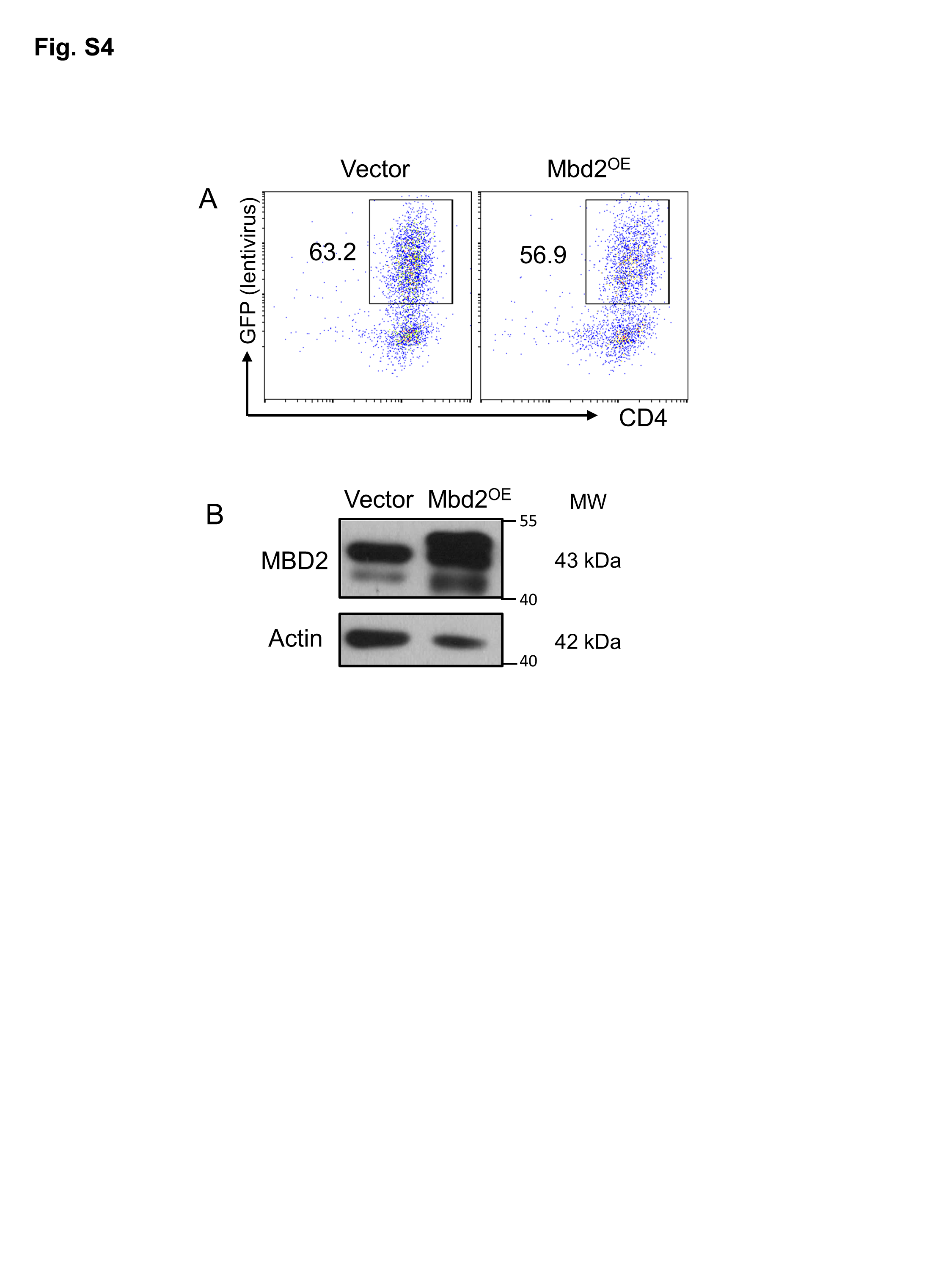

Supplement: Supplementary file 7 — Supplementary Figure 4. [file 41418_2021_852_MOESM7_ESM.tif]
